# Occupational exposures in low- and middle-income countries: A scoping review

**DOI:** 10.1371/journal.pgph.0003888

**Published:** 2024-11-15

**Authors:** Valentina Quintero Santofimio, Andre F. S. Amaral, Johanna Feary

**Affiliations:** 1 National Heart and Lung Institute, Imperial College London, London, United Kingdom; 2 NIHR Imperial Biomedical Research Centre, London, United Kingdom; PLOS: Public Library of Science, UNITED STATES OF AMERICA

## Abstract

Exposure to high levels of harmful agents in the workplace can significantly impact workers’ health, contributing to morbidity and mortality. Levels of these exposures are often measured in high-income countries in research studies and, in some places, to monitor levels in line with health and safety regulations. However, less is known about workplace exposure levels in low- and middle-income countries (LMICs). Our aim was to describe the quantitative exposure measurements of different occupational agents across industries within LMICs. We conducted a scoping review of peer-reviewed publications available on Web of Science and PubMed from inception to 1 September 2023. Our search focused on quantitative occupational exposure measurements across industries in LMICs. We identified a total of 8,676 publications. After screening, 58 studies from 25 countries were retained for final review. China, Iran, and Tanzania contributed the greatest number of studies. Manufacturing, mining, and agriculture were the most studied sectors, with factory workers and miners being the most common job titles. Exposure measurements included vapour, gases, dust, and fumes (VGDF), solvents, metals, pesticides and particulate matter. Occupational exposure levels for the same industry varied widely across geographical regions. This review provides a comprehensive overview of occupational exposures in LMICs and highlights the absence of data in certain geographical areas and industries. The study contributes valuable insights for directing future research, and the need to optimise the assessment of occupational exposures in LMICs with the aim ultimately of reducing disease.

## Introduction

Occupational exposures include agents encountered in the workplace and vary widely depending on the industry and job title [1]. Exposure to some agents have been previously linked to adverse health outcomes including cancer, respiratory diseases, and mortality [1–3]. The World Health Organisation (WHO) and International Labour Organisation (ILO) have estimated that, in 2016, 90 million disability-adjusted life years (DALYs) were attributable to occupational exposures, with the highest burden observed in the African region [4]. However, this estimate is based mostly on data collected in high-income countries (HICs), where several national databases of occupational exposures are available, and many primary studies have been conducted.

The ongoing world population growth, coupled with increasing production demands, is likely to increase morbidity and mortality in working populations, particularly where occupational exposures are unregulated [5]. Ensuring appropriate control of exposures in low- and middle-income countries (LMICs) is challenging due to more permissive and fewer policies regulating harmful exposures [6]. Occupational health is often neglected in LMICs and may be attributed to insufficient resources as well as poor data collection [7]. A large proportion of the literature on occupational exposure measurements originate from HICs and research from LMICs remains limited [8]. Workers in LMICs are likely to face higher levels of harmful agents compared to workers in HICs and, therefore, may experience higher risk of adverse health outcomes requiring targeted interventions [9]. The identification and accurate monitoring of occupational agents play an important role in workplace safety, aiming to mitigate risks, protect workers and promote a healthier work environment [10]. In addition, adequate safety measures including control of exposure to occupational agents and regulatory procedures are crucial to create a safer work environment [11]. Hence, it is imperative to understand better the scale of the problem in LMICs to try and protect workers and reduce burden of disease.

Common industries in LMICs include farming, manufacturing, and mining. Individuals working in these occupations are known to have high exposure to harmful agents such as vapours, gases, dust, and fumes (VGDF), metals and pesticides which may be detrimental to worker’s health [12, 13]. However, it is common for workers in LMICs to engage in informal “cottage” industries and artisanal production of wares, which may put them at high risk of exposures.

Research tools such as job-exposure matrices (JEM) are useful ways to assign occupational exposure to particular agents in epidemiological studies. These are based on job titles and eliminate the need to assess personal exposure levels, which are costly and often a challenge in LMICs. Disease-specific JEMs, have been developed. For example, the ALOHA+ JEM was developed to assign exposures of interest to chronic obstructive pulmonary disease (COPD) [12], while the SYN-JEM was developed with lung cancer in mind [14]. These, and other commonly used JEMs, were developed primarily for Europe and North America and are based on measurements from HICs [15]. Whether existing JEMs can be used in LMICs without leading to misclassification is unclear.

We conducted a scoping review to identify publications based in LMICs that reported occupational exposure measurements in different industries. Herein, we aimed to identify and report the quantitative exposure measurements of these agents. Additionally, this may serve as a starting point to direct further research on the applicability of JEMs to LMICs and whether calibration of existing JEMs would be beneficial using exposure levels reported within the literature.

## Methods

### Search terms and strategy

Our search strategy was developed in collaboration with an experienced science librarian. This scoping review was conducted following the guidelines highlighted by Peters et al (2020) [16]. It is reported in accordance with the Preferred Reporting Items for Systematic reviews and Meta-Analyses extension for Scoping Reviews (PRISMA-ScR) (S1 Table) [17]. This scoping review was guided by the research question “What is known from the existing literature about quantitative occupational exposure measures in LMICs?”. We searched Web of Science and Medline (PubMed) from database inception to 1 September 2023. We used text words and medical subject headings (MeSH) to identify publications with occupational exposure measurements in LMICs. The Cochrane Effective Practice and organisation of Care (EPOC) LMIC filters (February 2023) based on the World Bank yearly updated Country Classification were used in both databases [18]. We limited the search to studies in humans only and grey literature was not searched. The search terms are fully described in S2 Table.

### Screening and data extraction

We imported all identified publications into Covidence web-based software [19], and duplicates were automatically removed. Titles and abstracts of the records were screened and excluded if: [1] there were no quantitative exposure measurements; [2] measurements were not in an occupational setting; and [3] the study population were not adults. If there was insufficient information in the abstract, these were included in the full-text screening. Data were extracted using Covidence on study population characteristics, job title, industry, occupational agent, and quantitative measurements of personal or ambient exposure in the workplace. All the papers included were in English, except for two in Spanish and one in Polish, which we translated. All screenings and data extraction were conducted by VQS. Assessment of the quality of the included studies was beyond the scope of this review.

## Results

**Fig 1:**
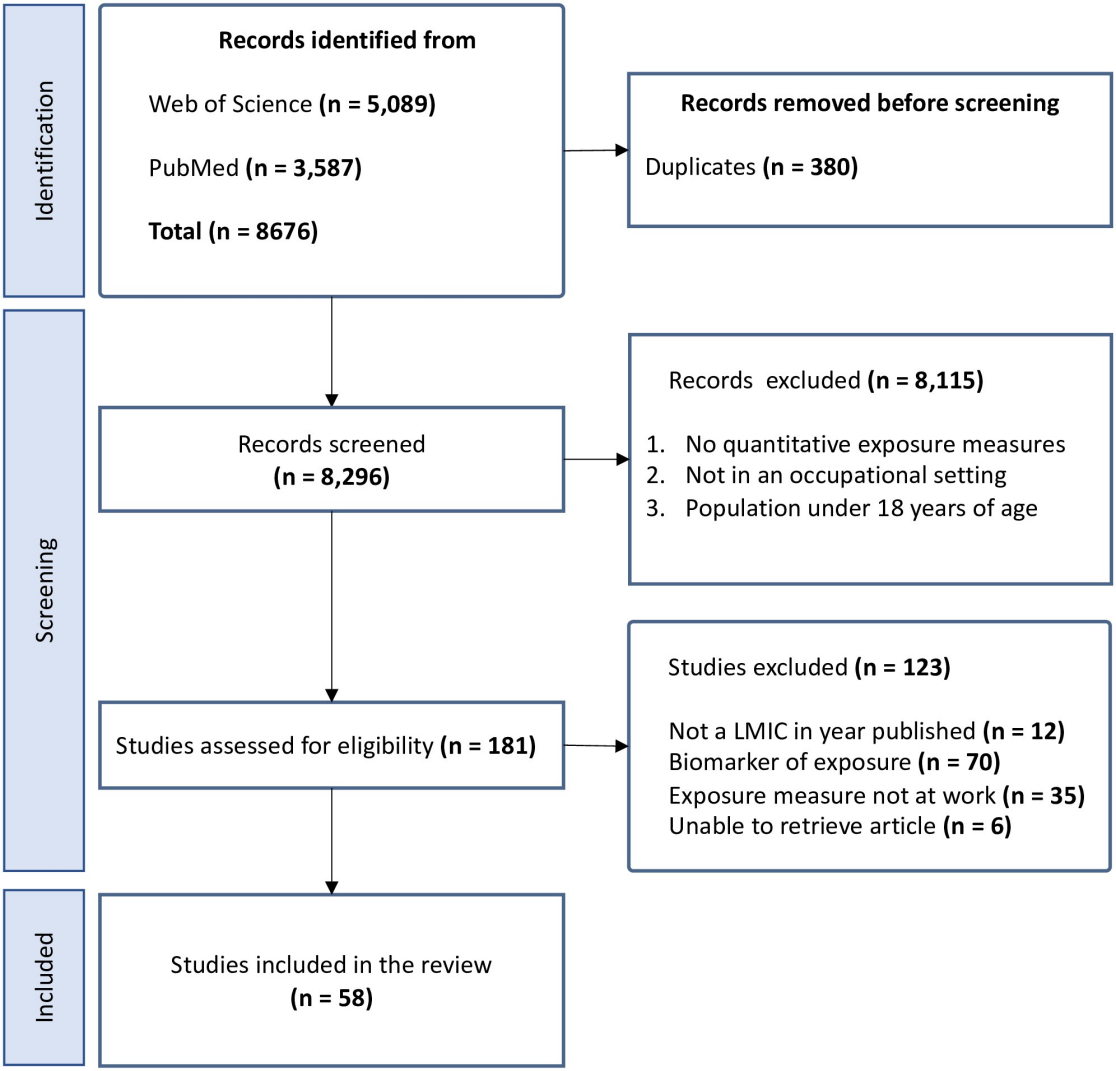
PRISMA flow diagram showing study selection.

We identified a total of 8,676 publications from the initial search, of which 181 were considered for full text review. A total of 58 publications were selected for inclusion, after excluding 123 full texts. The primary cause for exclusion was the absence of direct occupational exposure measurements, and instead using analysis of biomarkers to determine exposure. Additionally, some publications reported exposure measurements in other settings such as at home or in the local area instead of work based. Details of the selection are shown in Fig 1. An overall summary of the publications included is shown in Table 1.

**Table 1:** Summary of publications included in the review measuring occupational exposures to different agents.

Author, year	Study design	Exposed population (N)	Sex, male (%)	Unexposed population (N)	Job title	Country, WHO Region
**Mineral dust (N = 20)**						
Tefera et al. (2020) [20]	Cross sectional	630	630 (100%)	-	Textile factory workers	Ethiopia Africa
Sepadi et al. (2020) [21]	Cross sectional	34	29 (85.3%)	-	Platinum miners	South Africa Africa
Souza et al. (2021) [22]	Cross sectional	258	258 (100%)	-	Miners	Brazil Latin America
Wen et al. (2019) [23]	Cross sectional	98	97 (98.9%)	-	Rhinestone factory	China Western Pacific
Sanjel et al. (2018) [24]	Cross sectional	46	N/A	-	Clay brick workers	Nepal Southeast Asia
Rusibamayila et al. (2018) [25]	Cross sectional	112	107 (95.55)	-	Miners	Tanzania Africa
Sayler et al. (2018) [26]	Cross sectional	46	46 (100%)	-	Stone processing workers	Thailand Southeast Asia
Sanjel et al. (2016) [27]	Cross sectional	400	298 (74.5%)	400 (grocery workers within local area)	Brick kiln workers	Nepal Southeast Asia
Chen et al. (2014) [28]	Cross sectional	51	46 (90.2%)	41 (same factory, unexposed)	Industrial workers	China Western Pacific
Cely-Garcia et al. (2012) [29]	Cross sectional	6	6 (100%)	-	Mechanics	Colombia Latin America
Kakooei et al. (2011) [30]	Cross sectional	75	75 (100%)	-	Mechanics	Iran Eastern Mediterranean
Naghizadeh et al. (2011) [31]	Cross sectional	48	48 (100%)	-	Iron ore miners	Iran Eastern Mediterranean
Ehrlich et al. (2011) [32]	Cross sectional	520	N/A	-	Miners	South Africa Africa
Osman et al. (2009) [33]	Cross sectional	328	N/A	328 (store employees, unexposed)	Wood industry workers	Turkey Europe
Neghab et al. (2009) [34]	Cross sectional	33	33 (100%)	20 (males from the same industry with almost identical socioeconomic and demographic status (sex, ethnic background, education, smoking habits, income as well as family size), unexposed	Ceramic workers	Iran Eastern Mediterranean
Mamuya et al. (2006) [35]	Longitudinal	133	133 (100%)	8 (office workers within the same mine, unexposed)	Coal miners	Tanzania Africa
Mwaiselage et al. (2006) [36]	Cross sectional	51	51 (100%)	33 (office worker)	Cement workers	Tanzania Africa
Akkurt et al. (2006) [37]	Cross sectional	406	N/A	146 (same factory, unexposed)	Cement factory workers	Turkey Europe
Bratveit et al. (2003) [38]	Cross sectional	21	21 (100%)	-	Small-scale miners	Tanzania Africa
Kim et al. (2002) [39]	Cross sectional	49	N/A	88 (clinical laboratory or administrative workers, unexposed)	Dental technician	South Korea Western Pacific
**VGDF (N = 18)**						
Abdel-Rasoul et al. (2022) [40]	Cross sectional	110	110 (100%)	110 (age matched, unexposed)	Welders	Egypt, Eastern Mediterranean
Neghab et al. (2018) [41]	Cross sectional	100	100 (100%)	100 (age, sex matched)	Wood factory workers	Iran Eastern Mediterranean
Leon-Kabamba et al. (2018) [42]	Cross sectional	199	199 (100%)	242 (local administrative employees)	Coltan miners	Democratic Republic of Congo Africa
Black et al. (2017) [43]	Cross sectional	100	89 (89%)	17 (no exposed activities: excavating, crushing, milling, and concentrating)	Artisanal and small-scale gold miners	Burkina Faso Africa
Al-Zubaidi et al. (2017) [44]	Case control	30	N/A	5 (age matched, unexposed)	Dentist	Iraq Eastern Mediterranean
Nurul et al. (2016) [45]	Cross sectional	184	184 (100%)	-	Steel workers	Malaysia Southeast Asia
Hu et al. (2016) [46]	Cross sectional	97	59 (60.8%)	-	Petrol station workers	China Western Pacific
Tohon et al. (2015) [47]	Cross sectional	140	N/A	-	Gasoline workers	Benin Africa
Estevez-Garcia et al. (2013) [48]	Cross sectional	477	458 (96.1%)	97 (office police officers)	Traffic police officers	Colombia Latin America
Thetkathuek et al. (2010) [49]	Cross sectional	685	316 (46.1%)	Office workers within the factory but N not specified	Furniture factory workers	Thailand Southeast Asia
He et al. (2009) [50]	Cross sectional	325	325 (100%)	-	Bisphenol A (BPA) factory workers	China Western Pacific
Mamuya et al. (2007) [51]	Cross sectional	299	299 (100%)	49 (office workers within the same factory, unexposed)	Coal miners	Tanzania Africa
Xiao et al. (2006) [52]	Cross sectional	1709	788 (46.1%)	-	Factory workers	China Western Pacific
Hu et al. (2006) [53]	Cross sectional	712	N/A	211 (workers from the institute of equipment calibration)	Coke oven workers	China Western Pacific
Golbabaei et al. (2005) [54]	Cross sectional	51	6 (11.8%)	-	Dental technician	Iran Eastern Mediterranean
Tan et al. (2000) [55]	Cross sectional	514	N/A	202 (same factory, unexposed)	Viscose factory workers	China Western Pacific
Meneses et al. (1999) [56]	Cross sectional	46	N/A	-	Service station workers and street vendors	Mexico Latin America
Ho et al. (1998) [57]	Cross sectional	111	111 (100%)	111 (age and ethnicity matched)	Industry workers	China Western Pacific
**Biological dust (N = 7)**						
Rodriguez-Zamora et al. (2017) [58]	Cross sectional	136	136 (100%)	-	Grain storage workers	Costa Rica Latin America
Hamatui et al. (2016) [59]	Cross sectional	307	155 (50.5%)	-	Charcoal-processing workers	South Africa Africa
Chen et al. (2004) [60]	Cross sectional	197	39 (19.8%)	40 (similar labour intensity, unexposed)	Silk processing workers	China Western Pacific
Segvic Klaric et al. (2012) [61]	Longitudinal	96	96 (100%)	-	Farmer	Croatia Europe
Szadkowska-Stanczyk et al. (2010) [62]	Cross sectional	90	N/A	-	Swine farmers	Poland Europe
Mackiewicz et al. (1998) [63]	Cross sectional	53	N/A	53 (workers of an industrial factory (machinery industry, unexposed))	Swine farmers	Poland Europe
Shin et al. (2019) [64]	Cross sectional	-	N/A	-	Swine farmers	South Korea Western Pacific
**Solvents (N = 6)**						
Kouidhi et al. (2017) [65]	Cross sectional	70	N/A	70 (age matched within the same factory, unexposed)	Plastic industry worker	Malaysia Southeast Asia
Vargas-Ramos et al. (2014) [66]	Cross sectional	41	N/A	25 (same factory, unexposed)	Furniture factory workers	Colombia Latin America
Sekkal et al. (2012) [67]	Cross sectional	250	250 (100%)	250 (electricians, unexposed)	Petroleum product workers	Algeria Africa
Park et al. (2009) [68]	Cross sectional	41	23 (56.1%)	90 (males, from paper manufacturing company or hospital restaurant)	Industrial plant worker	South Korea Western Pacific
Azimi Pirsaraei et al. (2009) [69]	Cross sectional	179	179 (100%)	-	Dry-cleaning shops (machine operator, presser, and counter staff)	Iran Eastern Mediterranean
Tovalin-Ahumada et al. (2007) [70]	Cross sectional	35	35 (100%)	33 (office worker)	Street vendors	Mexico Latin America
**Metals (N = 4)**						
Darvishi et al. (2019) [71]	Cross sectional	59	9 (15.4%)	-	Compact fluorescent lamp factory worker	Iran Eastern Mediterranean
Singh et al. (2018) [72]	Cross sectional	20	N/A	-	Electronic waste recycling workers	India Southeast Asia
Ho et al. (1998) [57]	Cross sectional	111	111 (100%)	111 (age and ethnicity matched)	Industry workers	China Western Pacific
Ellingsen et al. (2006) [73]	Cross sectional	96	96 (100%)	96 (age matched, unexposed)	Welders	Russia Europe
**Particulate matter (N = 3)**						
Ngo et al. (2015) [74]	Cross sectional	12	12 (100%)	-	Street vendors, bus drivers, mechanics	Kenya Africa
Kongtip et al. (2012) [75]	Cross sectional	80	76 (95%)	-	Bus driver	Thailand Southeast Asia
Ingle et al. (2005) [76]	Longitudinal	60	N/A	60 (healthy volunteers in Jalgaon e.g bank workers, private institution)	Traffic police officers	India Southeast Asia
**Pesticides (N = 1)**						
Norkaew et al. (2021) [77]	Cross sectional	30	30 (100%)	-	Farmers	Thailand Southeast Asia

### Study characterisation by year of publication, country and WHO region

**Fig 2:**
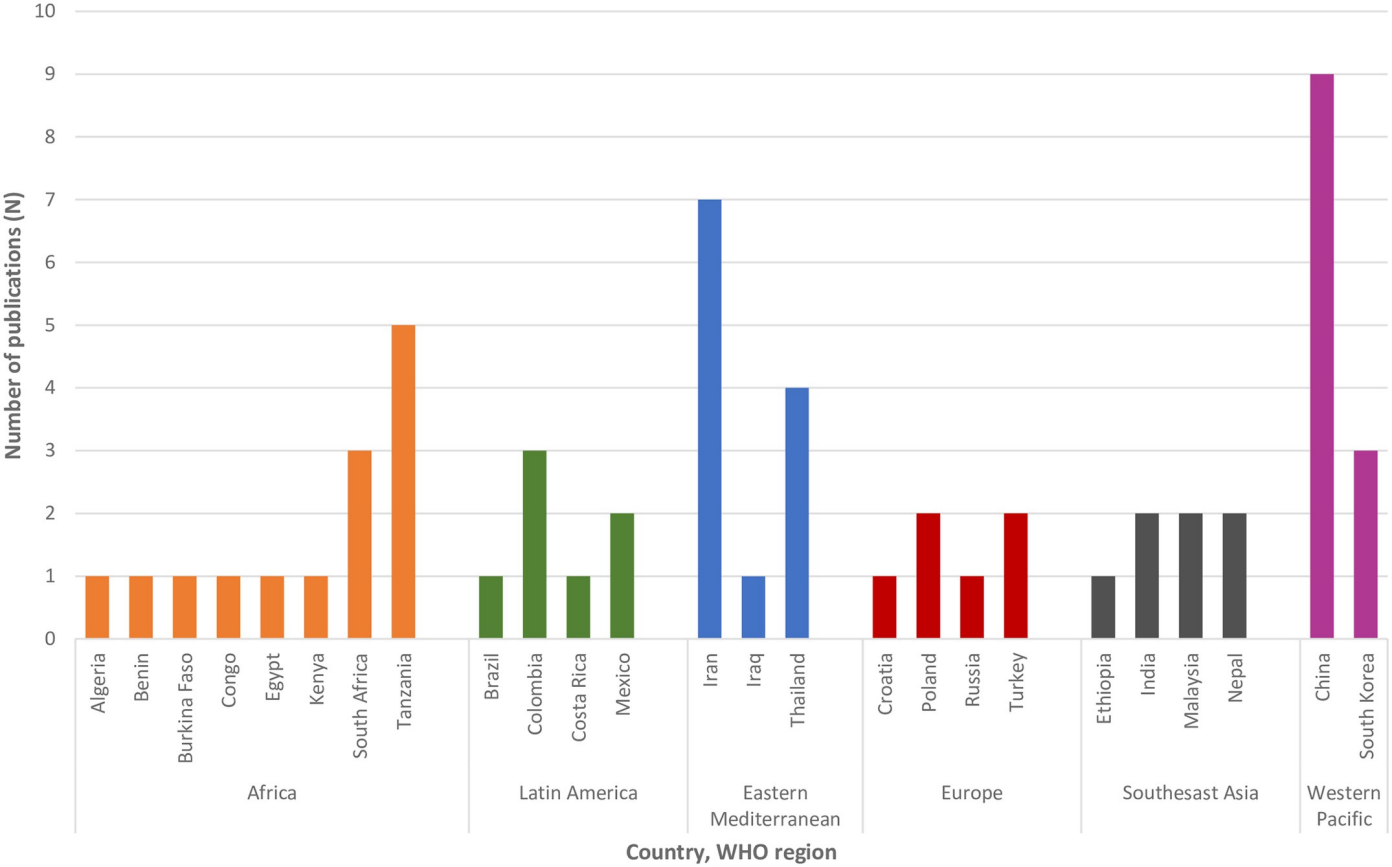
Publication counts by country, grouped by WHO region.

Fifty-four publications were cross-sectional studies, three were longitudinal studies and one was a case-control study (Table 1). The publication date of the studies ranged from 1998 to 2022. Over 60% (n = 36) were published between 2010 and 2022. The greatest number of publications by country were China (n = 9), followed by Iran (n = 7) and Tanzania (n = 5). The number of publications by country ranged from 1 to 9. When grouping countries by WHO region, similar numbers were from Africa (n = 14), Western Pacific (n = 12) and Southeast Asia (n = 10) regions (Fig 2).

### Study characterisation by occupational sector and job title

**Fig 3:**
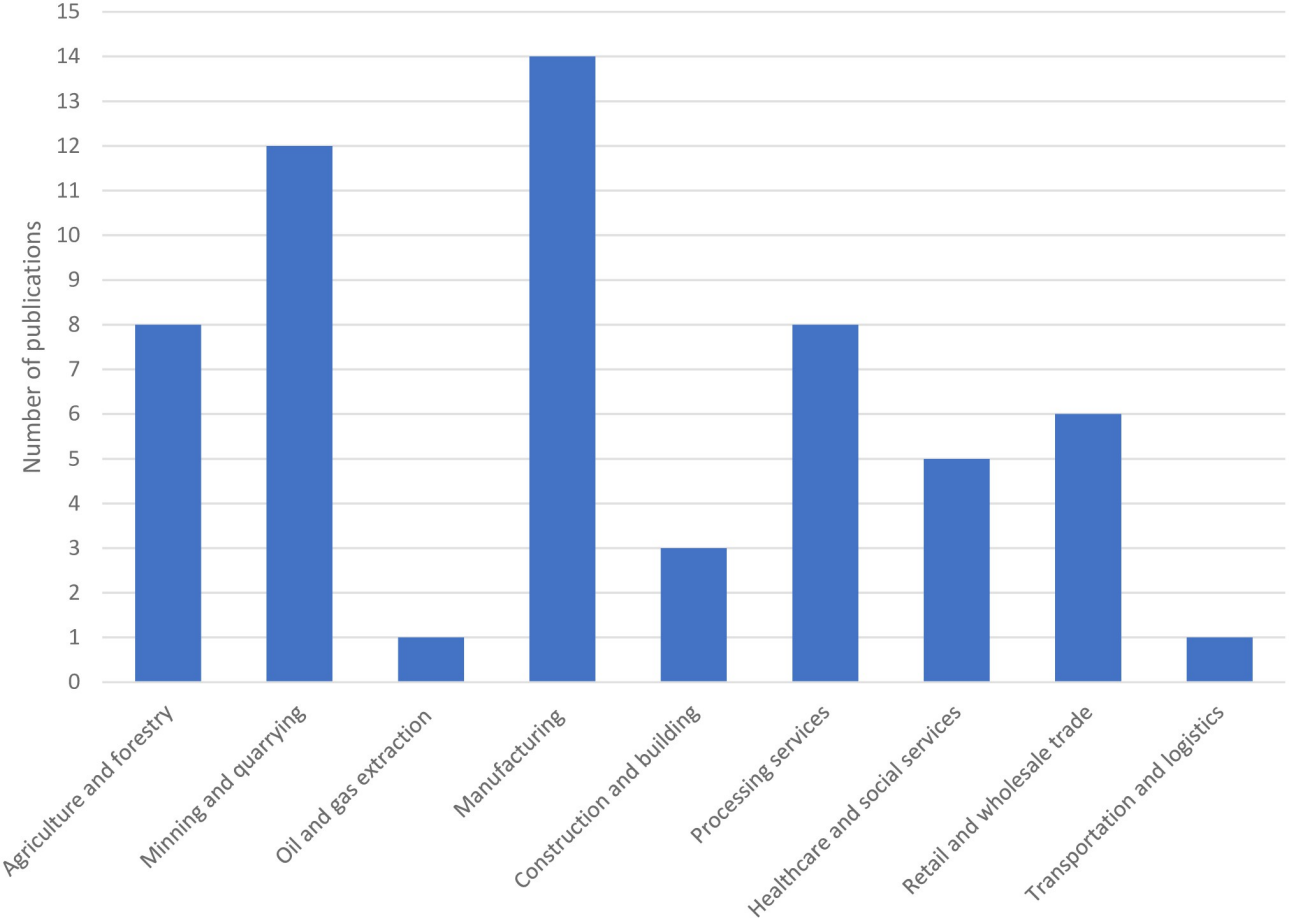
Publication counts by occupational sector.

Nearly 60% of the publications were based on the manufacturing (n = 14), mining and quarrying (n = 12) and agriculture and forestry (n = 8) sectors (Fig 3). The most common job titles were factory workers (n = 16) and miners (n = 11). Factory workers were employed across a diverse array of industries including textiles, wood, ceramics, plastics, solvents, and furniture manufacturing. Miners were involved in artisanal and small-scale gold, coltan and silica mining. One study was conducted in an illegal E-waste processing setting, while the rest were carried out in legal settings.

The sample size across the selected publications ranged from 6 to 1,709 participants, with a median of 98 (48–205). 52% (n = 30) of the studies were composed of less than 100 participants; 47% (n = 27) had over 100 participants in the study. One study only collected ambient exposure across different farms thus it did not include participants. Sex of participants was not reported in 29% (n = 17) of studies; 60.1% (n = 25) of the publications that reported information on sex, were composed of male participants only. The remaining studies included both males and females (n = 16). A total of 28 studies failed to report smoking status, and 36 studies did not provide any details on the use or availability of PPE. Studies with a control group primarily consisted of office workers who were sex- and age-matched or workers within the same facility without exposure. Most studies indicated an average employment time of greater than 10 years (n = 24), and an average daily working duration of 1–8 hours (n = 27). However, 15 studies (25.9%) did not provide information on employment time, and 25 studies (43.1%) did not report information on average working hours (Table 2).

**Table 2:** Distribution of publications by different characteristics.

Characteristics	Number of publications (n = 58)
**Study sample size**	98 (48–205)
(Median, IQR)	
**Sex**	
Males and females	16 (27.6%)
Females only	0
Males only	25 (43.1%)
Not reported	17 (29.3%)
**Smoking status**	
Yes	30 (51.7%)
No	28 (48.3%)
**Average employment time**	
0–5 years	10 (17.2%)
>5years	9 (15.5%)
>10 years	24 (41.4%)
Not reported	15 (25.9%)
**Average daily working hours**	
1–8 hours	27 (46.6%)
>8 hours	6 (10.3%)
Not reported	25 (43.1%)
**PPE available**	
Yes	15 (25.9%)
No	7 (12.1%)
Not reported	36 (62%)

### Study characterisation by occupational exposure

Occupational exposures were measured through various methods and included measurement of personal exposure (n = 27), ambient exposure (n = 28), or both (n = 3). Some publications followed the US National Institute for Occupational Safety and Health (NIOSH) standardised methods. The results were reported as either arithmetic mean or time-weighted average (TWA). The most common occupational exposure measure was mineral dusts (n = 20), followed by VGDF (n = 18), and biological dust (n = 7). The exposure levels reported by each publication are described in Table 3.

**Table 3:** Exposure levels by publication and occupational agent.

**Mineral dust (N = 20)**			
**Author, year**	**Exposure level (Exposed group)**	**Exposure level (Control group)**	**Sampling strategy**
Tefera et al. (2020) [20]	Inhalable dust 3 (0.1–10.5) Units: median (range) mg/m^3^	N/A	Personal inhalable cotton dust was collected for an average duration of 4.3 hours (3.8 = -5.8 hours) in the period of January to February 2017. A total of 96 samples were collected
Sepadi et al. (2020) [21]	Mineral dust 0.03 (2.2) Units: mean (SD) mg/m^3^	N/A	Static dust sampling in the crusher plants conducted in October 2018 for workers’ shifts length
Souza et al. (2021) [22]	Crystalline silica dust 1.6 (0.41) Crude dust 13.1 (0.55) Units: median (IQR) mg/m^3^	N/A	Personal air exposure
Wen et al. (2019) [23]	Total dust 0.98 (0.94) Units: mean (SD) mg/m^3^	N/A	Personal air exposure
Sanjel et al. (2018) [24]	Quartz concentrations 331 (233–471) Units: median (range) μg/m^3^ Total dust 0.44 (0.30) Units: mean (SD) mg/m^3^	N/A	Kilns ambient exposure on working days for a week e.g., a typical work was 6 days per week, up to 14 hours per day.
Rusibamayila et al. (2018) [25]	Underground workers 0.41 (0.28) Open pit workers 0.17 (0.23) Units: mean (SD) mg/m^3^	N/A	Personal respirable dust exposure for 8hrs
Sayler et al. (2018) [26]	Silica dust 5.7 (5.2) Units: mean (SD) mg/m^3^	N/A	Personal sampling pumps for air sampling.
Sanjel et al. (2016) [27]	Total dust 5.18 Units: mean mg/m^3^ PM1 3.95 PM2.5 3.96 PM10 4.96 Units: mean mg/m^3^	Total dust 0.09 Units: mean mg/m^3^ PM1 0.08 PM2.5 0.08 PM10 0.08 Units: mean mg/m^3^	The samples were taken by placing the DustTrak near to the workstations at the height of the workers breathing zone for 2hrs.
Chen et al. (2014) [28]	Blending 0.51 (0.002) Drying, sifting 1.18 (0.02) Weighting 1.18 (0.01) Double decomposition 0.42 (0.00) Units: mean (SD) mg/m^3^	0.2 mg/m^3^	Monitored consecutively for 2 days in 4 different locations.
Cely-Garcia et al. (2012) [29]	Total dust 0.189 (0.006–3.493) Units: median (range) mg/m^3^	N/A	Three or four consecutive days using personal sampling equipment
Kakooei et al. (2011) [30]	Car mechanics 0.92 (2.52) Truck mechanics 0.46 (2.57) Units: mean (SD) mg/m^3^	N/A	Personal air sampling
Naghizadeh et al. (2011) [31]	Total dust 801 (55) Units: mean (SD) mg/m^3^	N/A	Sampling was based on environmental and personal sampling methods for 4hrs and 2 hrs
Ehrlich et al. (2011) [32]	Total dust 8.2 (2.9) Quartz dust 1.15 (0.44) Units: mean (SD) mg/m^3^	N/A	5 day sampling for 8 hrs
Osman et al. (2009) [33]	Total dust 2.04 (1.53) Units: mean (SD) mg/m^3^	Total dust 0.0006 (0.00025) Units: mean (SD) mg/m^3^	Personal samples for 8hrs
Neghab et al. (2009) [34]	Total dust 71.7 (16.2) Units: mean (SD) mg/m^3^	Undetectable	Personal air sampling
Mamuya et al. (2006) [35]	Total dust 18.17 Units: mean mg/m^3^	Undetectable	Personal dust exposure was measured in two periods (1) June to August 2003 (2) July to August 2004
Mwaiselage et al. (2006) [36]	Total dust 4.0 Units: mean mg/m^3^	Undetectable	Personal exposure in the breathing zone was measured by sampling respirable dust (n = 30) and total dust (n = 15) among randomly chosen workers from the study population.
Akkurt et al. (2006) [37]	Asbestos fibre 0.22 fibre/cm^3^	Undetectable	Ambient sampling
Bratveit et al. (2003) [38]	Total dust 28.4 (12.4–29) Units: median (range) mg/m^3^	N/A	Personal dust sampling during the day shift on 3 consecutive working days.
Kim et al. (2002) [39]	Silica dust 6.51 (6.07) Total dust 14.88(11.2) Units: mean (SD) μg/m^3^	Undetectable	50 personal air sampling for 6 hrs
**VDGF (N = 18)**			
**Author, year**	**Exposure level (Exposed group)**	**Exposure level (Control group)**	**Sampling strategy**
Abdel-Rasoul et al. (2022) [40]	Total welding fumes 5.2 (0.77) Units: (mean, SD) mg/m^3^	N/A	Personal air-samples 8hrs/sample
Neghab et al. (2018) [41]	Factory fumes 6.76 (1.71) Units: (mean, SD) mg/m^3^	Total fumes 2.44 (0.66) Units: (mean, SD) mg/m^3^	Personal sampling
Leon-Kabamba et al. (2018) [42]	Mining fumes 197 (180–210) Units: (median, range) μg/m^3^	Undetectable	Mining fumes measured in workers and workstation (in offices for control sites), within an interval of 30 min, by means of Bramc Air quality monitor BRAIR-329
Black et al. (2017) [43]	Total mining fumes 7026 (6857) Mercury fumes 1412 (2870) Units: (mean, SD) mg/m^3^	Undetectable	Personal Hg vapour badge, during 3 different amalgam burn events on different days, for 1–4 hours
Al-Zubaidi et al. (2017) [44]	Mercury vapour S1 (lowest: 61.3, highest: 129.8) S2 (lowest: 143.8, highest: 938.0) S3 (lowest: 101.3, highest: 185.8) Units: (mean, SD) mg/m^3^	N/A	Seasonally from Feb to Nov 2016 (S1) Beside the amalgamator or the area around the capsule mixer (S2) Above the work surface (S3) Around patient’s chair
Nurul et al. (2016) [45]	Total vapour/gas Steel making plant 1.43 (0.04–12.59) Direct reduction plant 1.09 (0.06–16.35) Support staff 0.73 (0.02–17.95) Units: (median, range) mg/m^3^	N/A	8hrs average calculated from samples
Hu et al. (2016) [46]	Methyl tertiary butyl ether (MTBE) gases Operating workers 388.38 Support staff 71.61 Units: (mean) μg/m^3^	N/A	Ambient sampling
Tohon et al. (2015) [47]	Vapours Benzene *(unofficial sellers)* 478 *(Official sellers)* 79 Units: (mean) μg/m^3^	N/A	Breathing zone samplers
Estevez-Garcia et al. (2013) [48]	139.4 (76) Units: mean (SD) μg/m^3^	Undetectable	Personal monitoring during working hours
Thetkathuek et al. (2010) [49]	4.08 (1.42) Units: mean (SD) mg/m^3^	Undetectable	Ambient sampling
He et al. (2009) [50]	Resin gases 7.89 (1.55–55.2) Bisphenol A vapours 4.72 (2.09–6.06) Total VGDF 6.67 (1.61–37.8) Units: median (range) mg/m^3^	N/A	Personal samplers for 3 days for the whole shift duration
Mamuya et al. (2007) [51]	Cumulative VGDF exposure 37.7 (78.1) Cumulative quartz exposure 2.0 (3.8) Units: mean (SD) mg/yr/m^3^	Cumulative VGDF exposure 7 Cumulative quartz exposure 0.3 Units: mean (SD) mg/yr/m^3^	Personal air sampling measured in two periods (1) June to August 2003 (2) July to August 2004.
Xiao et al. (2006) [52]	20.00 (3.14) Units: mean (SD) mg/m^3^	N/A	Ambient sampling
Hu et al. (2006) [53]	Top of coke oven 743.8 Bottom of coke oven 210.5 Units: mean mg/m^3^	Undetectable	Personal air sampling for 4 consecutive days
Golbabaei et al. (2005) [54]	Methyl methacrylate (MMA) vapour 17.38 (16.60–18.08) Units: median (range) mg/m^3^	N/A	Air samples collected on 3-day-a-week investigation was conducted in each laboratory (Saturday, Monday, and Wednesday)
Tan et al. (2000) [55]	Filament workers 20.05 (1.33) Staples workers 13.72 (1.12) Units: mean (SD) mg/m^3^	Undetectable	Personal air sampling pumps for 2hrs for four days
Meneses et al. (1999) [56]	Service-station 359.5 (170.4) Street vendors 83.7 (45.0) Units: mean (SD) mg/m^3^	N/A	Ambient sampling
Ho et al. (1998) [57]	Factory fumes 0.036 (0–0.2770) Units: median (range) mg/m^3^	Undetectable	Personal sampling for two consecutive days for 6hr shift
**Biological dust (N = 7)**			
**Author, year**	**Exposure level (Exposed group)**	**Exposure level (Control group)**	**Sampling strategy**
Rodriguez-Zamora et al. (2017) [58]	Grain dust 2.0 (0.2–275.4) Units: median (range) mg/m^3^	N/A	Dust samplers across the farm for 7.5hrs 2–3 times
Hamatui et al. (2016)	Dust 16.4 (0.2–33) Units: median (range) mg/m^3^	N/A	Personal dust levels within the farm for eight-hour shift on average of four working days
Chen et al. (2004) [60]	Silk dust Selecting 5.1 Washing 2.6 Roving processing 3.8 Spinning 3.3 Units, mean mg/m^3^ Fungal levels Selecting 5,028 (140) Washing 3,665 (364) Roving processing 5,788 (320) Spinning 2,202 (203) Units: mean (SD), CFU/m^3^	Undetectable levels	Ambient exposure monitored consecutively for two days at four different locations.
Segvic Klaric et al. (2012) [61]	Airborne fungi Area 1 1,696–7,316 Area 2 1,706–4,819 Units: mean (SD), CFU/m^3^	N/A	Over a one-year period at two month intervals from April 2008 to February 2009 at different sites within the sawmills.
Mackiewicz et al. (1998) [63]	Farming dust 8.76 (3.03–14.05) mg/m^3^ Units: median (range) mg/m^3^	Undetectable	Air samples were collected in the very centre of an examined piggery, at a height of 145 cm
Szadkowska-Stanczyk et al. (2010) [62]	Farming dust 5.38 (5.41) Units: mean (SD) mg/m^3^	N/A	
Shin et al. (2019) [64]	Farming dust 0.505 (0.354) Units: (mean, SD) mg/m^3^	N/A	The dust in each farm building was sampled for 6 hr, from 9 AM until 3 PM, at two different locations
**Solvents (N = 6)**			
**Author, year**	**Exposure level (Exposed group)**	**Exposure level (Control group)**	**Sampling strategy**
Kouidhi et al. (2017) [65]	Bisphenol A (BPA) 16.20 (9.87) Units: (mean, SD) mg/m^3^	BPA 3.10 (1.52)	Ambient sampling
Vargas-Ramos et al. (2014) [66]	Benzene 9.5 (2.9) Toluene 8.1 (1.0) Units: (mean, SD) mg/m^3^	Benzene 0.2 (0.08) Toluene 0.3 (0.15) Units: (mean, SD) mg/m^3^	Ambient sampling
Sekkal et al. (2012) [67]	Fuel loader Benzene 0.4 (0.02–1.7) Methyl-butane 3.1 (1–9.5) Pentane 3.0 (1–8.2) Hexane 1 (1–3.4) Brush painter Benzene 2.5 (0.7–12.8) Toluene 6.9 (1–44) Ethyl-benzene 4.0 (1–23.9) 1,2,4-Trimethyl-benzene 22.7 (1–75.2) Pentane 9.7 (4.7–31.0) Hexane 5.4 (1–25.2) Units: mg/m^3^ (median, range)	Electricians Undetectable levels	Ambient exposure in two periods April to June 2009 and January to March 2010.
Park et al. (2009) [68]	Mean (SD) 0.47 (0.33) mg/m^3^	Undetectable levels	Ambient exposure measurements during work shift
Azimi Pirsaraei et al. (2009) [69]	Machine operators 11.5 (16.9) Pressers 9.6 (20.4) Counter staff 7.2 (11.9) Units: (mean, SD) mg/m^3^	N/A	8-h time weighted average (TWA) air sample. NIOSH Method 1003
Tovalin-Ahumada et al. (2007) [70]	Benzene 59 (101) Toluene 256 (305) Units: (mean, SD) mg/m^3^		Personal exposure from April to May 2002, for a total of 18 days, during week-days.
**Metals (N = 4)**			
**Author, year**	**Exposure level (Exposed group)**	**Exposure level (Control group)**	**Sampling strategy**
Darvishi et al. (2019) [71]	Mercury vapour 15.90 (6.42) Range: 23–175 Units: mean (SD), μg/m^3^	N/A	Sampling for 8/hrs at different fixed positions 1.5m above floor level
Singh et al. (2018) [72]	Air exposure* As = 19.4 (10.6) Ba = 529.6 (132.5) Cd = 4.4 (2.9) Co = 8.9 (4.5) Cr = 131.0 (65.3) Cu = 1564.1 (683.7) Pb = 819.1 (289.3) Ni = 89.0 (46.6) Zn = 2044.9 (614.9) Soil exposure* As = 39.9 (25.9) Ba = 976.4 (280.2) Cd = 8.3 (5.2) Co = 19.4 (9.4) Cr = 287.2 (110.8) Cu = 14543.4 (4593.2) Ni = 130.2 (35.3) Pb = 1615.8 (660.8) Zn = 4737.7 (1016.5) Units: mean (SD) μg/m^3^	N/A	1) Soil samples of the ground where recycling was being done 2) Dust from the platform where recycling activities were done
Ellingsen et al. (2006) [73]	Mn = 97 (3–4620) Fe = 894 (106–20300) Cr = 13 (1–976) Ni = 15 (1–270) Pb = 2.3 (0.9–79) Al = 57 (12–2280) Cu = 11 (2–168) Zn = 19 (1–2490) Mg = 17 (3–1580) Ti = 15 (1–787) Ca = 215 (26–8630) Si = 190 (25–3460) Na = 89 (2–8380) K = 75 (5–9880) Units: median (SD) mg/m^3^	Undetectable	Full-shift air samples were collected for two days with a total of 188 full-shift air samples
Ho et al. (1998) [57]	Lead 0.089 Units: mean μg/m³	Undetectable	Lead-in-air monitoring for the lead-exposed workers was conducted by personal sampling. Total of two consecutive samples for 6hr shift
**Particulate matter (N = 3)**			
**Author, year**	**Exposure level (Exposed group)**	**Exposure level (Control group)**	**Sampling strategy**
Ngo et al. (2015) [74]	PM2.5 Bus driver 103.8 (28.3) Mechanic 57.8 (18.4) Street vendor 69.7 (21.6) Black carbon Bus driver 63.9 (18.6) Mechanic 26.0 (8.3) Street vendor 30.0 (12.7) Units: mean (SD), μg/m^3^	N/A	8-hr average occupational exposure levels of fine particulate matter (PM2.5), black carbon. Personal sampling three days throughout the week for 8 h each day.
Kongtip et al. (2012) [75]	Air-conditioned buses 208.42 (87.41) Non-air-conditioned buses 322.01 (157.97) Units: mean (SD), μg/m^3^	N/A	Personal monitoring during working hours
Ingle et al. (2005) [76]	SOx 64 NOx 58 PM10 515 Units: mean (SD), μg/m^3^	Not reported	Personal air sampling during working shift
**Pesticides (N = 1)**			
**Author, year**	**Exposure level (Exposed group)**	**Exposure level (Control group)**	**Sampling strategy**
Norkaew et al. (2021) [77]	Pesticide (unspecified) 350 ppm	N/A	Personal air sampling during working shift

### Mineral dust

Approximately 35% (n = 20) of the selected publications reported exposure measurements of mineral dust, primarily collected from mining or factory-related activities. The levels of mineral dust exposure ranged from 0.03 to 801 mg/m^3^ across these publications.

In the African region, Tefera et al. (2020) reported median personal inhalable exposure to cotton dust in textile factory workers in Ethiopia, of 3 (IQR 0.1–10) mg/m^3^ from 96 samples. Rusibamayila et al. (2018) found higher mean personal respirable dust exposure in underground (0.4 mg/m^3^) compared with open pit (0.2 mg/m^3^) mine workers in Tanzania, revealing higher mean exposure in underground workers. Sepadi et al. (2020) reported low mean levels of total dust exposure (0.03 mg/m^3^) from mines in South Africa. Kakooei et al. (2011) found higher mean exposure levels among car mechanics (0.9 mg/m^3^) compared with truck mechanics (0.5 mg/m^3^). Ehrlich et al. (2011) reported mean total dust (8.2 mg/m^3^) and quartz (1.1 mg/m^3^) dust exposure in South African miners. In Tanzania, Mamuya et al. (2006) reported a mean total mineral dust exposure of 18 mg/m^3^ in coal miners. Similarly, Bratveit et al. (2003) reported a median exposure of total dust of 28 (IQR 12–29) mg/m^3^ in small-scale miners. In cement workers, Mwaiselage et al. (2006) measured a mean total dust exposure of 4.0 mg/m^3^.

In Asia, two studies conducted in Nepal by Sanjel et al. (2016) measured exposure levels in a stone processing industry, reporting a mean total dust of 5.2 mg/m^3^. Sanjel et al. (2018) showed a median exposure level to quartz of 331 (IQR 233–471) μg/m^3^ and a total dust mean level of 0.4 mg/m^3^ among clay brick workers. Sayler et al. (2018) registered a mean exposure of 5.7 mg/m^3^ of silica dust among stone-processing workers in Thailand.

In Latin America, Souza et al. (2021) measured median personal air exposure to crystalline silica dust in Brazil of 1.6 mg/m3 and to total dust of 13 mg/m^3^. Cely-Garcia et al. (2012) measured mechanics’ exposures to mineral dusts (0.19 mg/m^3^) in Colombia.

In the Eastern Mediterranean, Naghizadeh et al. (2011) reported a mean total dust exposure of 801 mg/m^3^ in iron ore miners in Iran. Neghab et al. (2009) reported total dust exposures of 72 mg/m^3^ in ceramic workers, Osman et al. (2009) compared exposure levels in exposed and unexposed staff within a wood factory in Turkey, revealing higher mean total dust exposure in exposed workers (2.1 mg/m^3^) compared to unexposed staff (0 mg/m^3^). Asbestos fibre concentration in cement workers in Turkey was reported by Akkurt et al. (2006) at 0.22 fibres/cm^3^. These findings are summarised in Table 3.

In Western Pacific, Wen et al. (2019) reported a mean total dust of 0.98 mg/m^3^ among rhinestone factory workers in China, showing a. Chen et al. (2014) monitored exposures in industrial workers over two consecutive days, reporting the highest mean total dust levels at 1.2 mg/m^3^ in the weighting and drying stations, significantly higher than the control group (0.02 mg/m^3^). Kim et al. (2002) measured reporting mean values of 6.5 mg/m^3^ and 15 mg/m^3^, of silica dust and total dust levels respectively among dental technicians.

### VGDF

A total of 18 studies provided quantitative measurements of VGDF ranging from 0.04 to 7,026 mg/m^3^, and were based on industrial processing activities, mining fumes, petrol station fumes, or welding fumes. The majority of these studies were conducted in the Western Pacific, Eastern Mediterranean, and African region.

In the African region, Leon-Kabamba et al. (2018) measured mining fumes among coltan miners, who had a median exposure of 197 mg/m^3^. Another study by Black et al. (2017) also measured total mining fumes but reported a much higher mean level of 7,026 mg/m^3^ and specifically mercury fumes of 1,412 mg/m^3^. Tohon et al. (2015) investigated the differences in VGDF in official and unofficial gasoline sellers, finding unofficial sellers had a 6-times higher exposure compared to official sellers (478 μg/m^3^ vs 79 μg/m^3^). Mamuya et al. (2007) measured longitudinal exposures in coal miners and reported cumulative exposure to VGDF and quartz in the exposure group were 38 mg/year/m^3^ and 2.0 mg/year/m^3^, respectively. The control group, composed of office workers, had a cumulative exposure to VGDF and quartz of 7 mg/year/m^3^ and 0.3 mg/year/m^3^, respectively.

In Latin America, Estevez-Garcia et al. (2013) compared exposure to VGDF between traffic police officers (139 μg/m^3^) and office-based police officers (undetectable) in Colombia. Meneses et al. (1999) reported higher mean ambient exposure levels to VGDF in service station workers (360 mg/m^3^) compared with street vendors (of 84 mg/m^3^) in Mexico..

In Asia, a single study by Thetkathuek et al. (2010) reported mean levels of fumes among furniture factory workers in Thailand of 4.1 mg/m^3^.

In the Eastern Mediterranean, Abdel-Rasoul et al. (2022) reported exposure levels to welding fumes of 5.2 mg/m^3^. Neghab et al. (2018) reported exposure to factory fumes by job titles, with exposed workers had a mean level of 6.8 mg/m^3^ compared to unexposed staff with a mean of 2.4 mg/m^3^. Mercury (Hg) fumes were measured in a study by Al-Zubaidi et al. (2017) across three different areas at four dental clinics in Iraq. Levels of Hg vapour in one of these clinics was consistently highest across all three measurement areas: [1] beside the amalgamator or the area around the capsule mixer, [2] above the work surface, [3] around patient’s chair cross all clinics: 130 mg/m^3^, 938 mg/m^3^, and 186 mg/m^3^ respectively. Golbabaei et al. (2005) reported the mean exposure to methyl methacrylate (MMA) vapours in three different laboratories as 17 mg/m^3^.

In the Western Pacific region, Hu et al. (2016) reported ambient exposure levels in petrol station workers with different job titles; operating workers had the highest mean vapour exposure (388 μg/m^3^) and support staff the lowest (72 μg/m^3^). He et al. (2009) studied personal exposure levels to three different agents in a Bisphenol A factory, with median levels for resin gases at 7.9 (IQR 1.6–55) mg/m3, BPA vapours at 4.7 (IQR 2.1–6.1) mg/m^3^, and total VGDF at 6.7 (IQR 1.6–38) mg/m^3^. Xiao et al. (2006) reported VGDF exposure of 20 mg/m^3^ among factory workers. Hu et al. (2006) reported VGDF exposure among factory workers, exploring variations across two different areas—on top of the coke oven and bottom of the coke oven. The mean exposure was higher at the top of the coke oven (744 mg/m^3^) compared with the bottom (210 mg/m^3^). Tan et al. (2000) investigated filament and staples workers in a viscose factory in China and found filament workers had a higher VGDF exposure of 20 mg/m^3^ compared to staples workers with a mean exposure of 14 mg/m^3^. Ho et al. (1998) reported median VGDF exposure levels of 0.04 (0–0.28) mg/m^3^ among factory workers in China. Nurul et al. (2016) reported total fume levels across three different job titles in a steel factory in Malaysia, with the steel making plant workers having a median exposure of 1.4 (IQR 0.04–13) mg/m^3^, and support staff a median of 0.73 (IQR 0.02–18) mg/m^3^.

### Biological dust

Biological dust exposure comprises of airborne particles originating from living organisms or their activities. The levels of biological dust ranged from 0.5 to 16 mg/m^3^ across the studies. Two publications, from South Korea and Croatia measured fungi exposure levels, which ranged from 1,696 to 5,788 CFU/m^3^. In the African region, Hamatui et al. (2016) reported a median biological dust exposure of 16 (0.2–33) mg/m^3^ among charcoal-processing workers in South Africa. In the Latin America region, Rodriguez-Zamora et al. (2017) measured personal exposure to grain dust in various areas of a farm in Costa Rica, reporting a median of 2.0 (IQR 0.2–275) mg/m^3^ in charcoal-processing workers.

In the Western Pacific, Chen et al. (2004) investigated silk dust and fungi levels across different job titles within a South Korean factory. The selecting station, involved in picking raw silk, showed the highest mean levels of silk dust. Additionally, the roving processing area showed the highest fungal levels with a mean of 5,788 CFU/m^3^. Shin et al. (2019) investigated exposure levels among swine farmers, reporting a mean ambient dust exposure of 0.5 mg/m^3^.

In Europe, Segvic Klaric et al. (2012) also measured airborne fungi exposure over one year at two-month intervals in a sawmill in Croatia with levels varying from 1,696–7,316 CFU/m^3^ (Table 3).

There were two farming studies based in Poland, Europe; the first by Mackiewicz et al. (1998), reported a median exposure to farming dust of 8.8 (3.0–14) mg/m^3^. The second by Szadkowska-Stanczyk et al. (2010) found mean farming dust exposure among swine farmers to be 5.4 mg/m^3^.

### Solvents

Measurement of solvents in studies included benzene, Bisphenol A (BPA), and a combination of volatile organic compounds (VOC) mainly associated with factory workers or petrol station assistants. Six publications reported these exposures with levels ranging from 0.5 to 256 mg/m^3^.

In Latin America, Vargas-Ramos et al. (2014) reported exposures to solvents in Colombian furniture factory workers. Exposed workers showed a mean benzene level of 9.5 mg/m^3^, compared to 0.2 mg/m^3^ in the control group and mean toluene exposure of 8.1 mg/m^3^ and 0.3 mg/m^3^ respectively. Tovalin-Ahumada et al. (2007) found exposure levels of benzene (59 mg/m^3^) and toluene (256 mg/m^3^) in Mexican street vendors, with a mean of and respectively. Sekkal et al. (2012) measured solvent exposure in two job titles within the same petroleum-product factory during two distinct periods. Fuel loaders exhibited the highest median exposure to methyl-butane at 3.1 mg/m^3^, while brush painters had the highest median exposure to 1,2,4-Trimethyl-benzene at 23 mg/m^3^. No detectable levels of any targeted agent were found among the control group of electricians (Table 3).

In the Eastern Mediterranean, Azimi Pirsaraei et al. (2009) investigated unspecified solvent exposure within a dry-cleaning shop in Iran and found machine operators had the highest mean exposure (11 mg/m^3^) and counter staff had the lowest exposure (7.2 mg/m^3^).

In the Western Pacific region, Kouidhi et al. (2017) measured ambient exposure of BPA in a plastic factory in Malaysia. This publication reported a mean level of 16 mg/m^3^ in areas directly involved in plastic processing, contrasting with levels of 3.1 mg/m^3^ among workers in the factory without direct duties, such as administrative or sales staff. Park et al. (2009) reported a mean ambient exposure to unspecified solvents of 0.5 mg/m^3^ in industrial plant workers in South Korea.

### Metals

Four publications provided insights into metal exposure, with levels ranging from 0.089 to 4,738 μg/m³. Ho et al. (1998), in addition to VGDF exposure, reported a median lead air concentration of 0.09 μg/m³ within plastic-processing industries in China. In the Eastern Mediterranean region, Darvishi et al. (2019) reported a mean mercury vapor level of 16 μg/m³ across various areas within a compact fluorescent lamp factory in Iran.

In Asia, Singh et al. (2018) investigated air and soil samples from an illegal electronic waste (E-waste) region in India, showing higher soil concentrations compared to air samples across various metals with zinc (Zn) levels being the highest (mean of 4,738 μg/m³). In a study by Ellingsen et al. (2006) based in Europe, exposure to different metals in air samples was measured over two consecutive full-shift days among welders. The highest and lowest mean concentrations were observed for iron (Fe) at 894 μg/m³ and lead (Pb) at 2.3 μg/m³, respectively. All recorded exposure levels exhibited considerable variability, as highlighted in Table 3.

### Pesticides

Only one study reported measurements of pesticides exposures. The exposure level to different classes of pesticides was 350 parts per million (ppm) collected through personal air sampling from rice farmers in Thailand, Southeast Asia region. The workers were involved in different activities ranging from the application and handling of pesticides and harvesting of rice crops, and exposure levels were the same across all job titles.

### Particulate matter

Particulate matter (PM) was measured in three publications, with all studies focusing on ambient exposures ranging from 104 to 515 μg/m^3^. In the African region, Ngo et al. (2015) conducted a study measuring exposure levels to PM2.5 and black carbon among bus drivers, mechanics, and street vendors in Kenya. Sampling was carried out personally over three days each week, spanning 8 hours per day. Bus drivers exhibited the highest mean PM2.5 and black carbon levels at 104 μg/m^3^ and 64 μg/m^3^, respectively, compared to other job titles (Table 3).

In the Southeast Asian region, Kongtip et al. (2012) investigated PM2.5 exposure among bus drivers operating air-conditioned or non-air-conditioned buses in Thailand. Higher exposure levels were observed among drivers of non-air-conditioned buses, with a mean of 322 μg/m^3^. Ingle et al. (2005) conducted a longitudinal study to assess the levels of emission particles alongside PM10, such as sulphur and nitrogen oxides. The highest mean levels were recorded for PM10 at 515 μg/m^3^ (Table 3).

## Discussion

A total of 58 studies from 25 countries across all WHO regions were identified in our review. The most common occupational agent category was VGDF, which was measured in all world regions and across different industries with significant variations in reported measurements in the same industry and world regions.

The publications in this review were mainly comprised of working male participants, highlighting the gender disparities in working populations in LMICs. Traditionally, in LMIC males dominate the labour markets, whereas females would assume a more traditional role e.g. domestic work [78]. The proportion of female workers is notably lower in some world regions such as the Middle East, North Africa, and South Asia [79]. Furthermore, where females do work, they are more likely to engage in unprotected ‘cottage’ jobs where occupational exposure may be most harmful [78, 79]. The female workers included in this review were primarily petrol station workers, artisanal and small-scale gold miners, charcoal-processing workers and dental technicians with high exposure levels to different occupational agents. However, given the predominance of male participants in the included publications, it would be difficult to generalise the findings to establish associations between occupational exposures and health outcomes in females. Therefore, more research focusing on female workers in various occupational settings is needed to better understand and mitigate the specific health risks they may face.

In this review we identified traditional industries such as manufacturing, mining and quarrying, and agriculture and forestry to be the most common occupational sectors. The most common job titles were miners and factory workers. The manufacturing sector included industries such as textiles, wood, ceramics, and furniture representing a wide variety. However, globally, and particularly in the African and Asian regions, the emergence of e-waste recycling is becoming of increasing concern and the paucity of published papers does not necessarily represent the scale of the problem. E-waste recycling is often performed in unregulated settings, and accounts for a large proportion of exposure to different metals such as lead [6]. A review by Parvez et al. (2021) estimated that over 74.7 million metric tons of e-waste will be produced globally by 2030, with over 80% of this waste, produced in HICs, illegally exported to LMICs, due to lower labour cost and poorly enforced laws [80]. In LMICs, e-waste recycling is conducted without modern technology, in informal settings, regardless of legislations to restrain illegal processing [81]. We identified one study based on an illegal e-waste setting in India, Southeast Asia by Singh et al. (2018). This study reported very high levels of individual metal exposure, such as lead air exposure (819.1 mg/m^3^) and zinc air exposure (2044 mg/m^3^) above the regulatory thresholds. For example, the recommended lead and zinc air exposure limit in India are 0.15 mg/m^3^ and 10 mg/m^3^ respectively [82]. E-waste recycling results in occupational exposure to harmful agents such as toxic metal chemicals for workers. However, on a larger scale incorrect disposal and processing on the waste can contaminate food, ground water reservoirs and air quality in surrounding areas [80] and this becomes of particular concern as early exposure (e.g. in utero or childhood) can cause devasting health consequences [83]. Likewise, there is increased focus on occupational exposure to micro-plastic (MP) and nano-plastics (NP). Once considered to be inert and non-toxic, emerging evidence now shows detrimental health effects of MP/NP, on cancer risk, respiratory health and neurodegeneration [84]. Furthermore, while PM exposure is widespread in the general public (for example, from traffic exhaust emissions) and may not be perceived to be an occupational exposure, some occupational groups, such as bus drivers, experience prolonged and consistent exposure during their working hours (in contrast, with passengers) and therefore we chose to include the relevant papers in this review.

We observed substantial variability and a wide range of exposure levels to the same agent across the included publications. This variability persisted even when categorising studies by WHO region, revealing significant discrepancies in reported levels among studies with similar working populations, such as miners or same-type factory workers. It is possible this variability may be reflecting differences in industrialisation, regulatory frameworks and time periods [6]. Equally, this review highlights the lack of standardised methodologies in conducting occupational exposure research. Sampling strategies are important for accurately reflecting true occupational exposure levels. For example, the duration of monitoring (e.g., one day vs. an average workday across multiple measurements) and the representativeness of the sampled data pose challenges. Utilising more robust measures, such as occupational hygiene reports over extended periods or longitudinal studies such as Mamuya et al. (2007) and Segvic Klaric et al. (2012) [51, 61] to estimate cumulative exposure, may offer better insights for accurately assigning exposure levels in different industries. The results from this scoping review can improve the understanding of the variations in exposure levels, and how they are likely to be higher than those encountered in HICs. This is crucial to help develop targeted interventions and improve research tools such as JEMs. This might include addition of missing occupational agents, job titles (from new emerging industries) or extra (higher) levels of exposure to existing JEMs to make them more representative of occupational exposures encountered in LMICs. Updating and adaption of JEMs ensures that research methodologies remain robust, particularly in large epidemiological studies. Thus contributing to better investigation of the impact of workplace exposures in health outcomes [15]. This will in turn aid with tailoring of occupational health and safety guidelines addressing workers’ challenges and promoting safer work environments across industries in LMICs. The timeline of publication dates in our review ranged from 1998 to 2022, peaking in 2006. Whilst over 60% (n = 35) of the publications occurred between 2010 and 2022, only 14 studies were published in the last five years. Many LMICs had no publications, although it may be possible that studies have been carried out and remain unpublished where similar levels may have been measured. In our initial search we identified over 8,000 publications; however, a large proportion did not report quantitative measurements, and instead used biomarkers to indicate possible exposures. Overall, we found very few recent publications which illustrates the challenges in addressing occupational exposures in LMICs particularly given the likely size of the population at risk. We acknowledge that there are a large number of industries and potential exposures where no exposure measurements have been reported at all and will not be included here. The reasons for this are multiple and include that conducting accurate exposure measurements which may require sophisticated monitoring equipment, data analysis tools, and training programs may not be possible in LMICs where funding is limited and where occupational health may not be a priority [85]. Future research efforts should aim to explore strategies to promote research initiatives in the field of occupational health, ensuring a sustained and global focus on the workers’ well-being.

Overall, this review with a good geographical distribution and which highlighted the need to better understand occupational exposure levels across different LMICs. We also observed a good representation of common industries in LMICs, such as manufacturing and mining industry and identified papers from emerging industries of importance. However, limitations include the lack of publications reporting exposure levels of pesticides, particularly given that agriculture is a prominent sector in LMICs. Additionally, the non-standardised exposure methodologies employed makes comparison between studies difficult and can result in misclassification of exposure levels. Finally, the absence of weighted assessments, the quality of the studies that we did not assess within this review, and varying sample size should be considered. Despite the limitations, the review represents an initial step forward in understanding occupational exposure measurements in LMICs, providing valuable insights into common industries and emphasising the need for future research.

## Conclusion

In conclusion, this review provides an insight of the landscape of occupational exposures in LMICs. The findings reveal a general paucity of data in many geographical regions and industries. Whilst it is difficult to draw direct comparisons between industries and countries, it is clear that exposure levels are generally high, and any variation may reflect differences in study methodologies or work environments. This review lays a foundation to encourage future research in LMICs to accurately report occupational exposures. This is important to ensure risks relating to occupational exposures are identified and tools such as JEMs are adapted if necessary to reflect exposures that occur in LMIC. Ultimately, the aim is to mitigate adverse health outcomes resulting from high exposure levels in working populations in developing countries.

## Supporting information

S1 Table PRISMA checklist for scoping reviews. (DOCX)

S2 Table Search terms used within Web of Science and Medline (PubMed). (DOCX)
