# Supplementary material for: Occupational exposures in low- and middle-income countries: A scoping review
Source: PLOS Glob Public Health. 2024 Nov 15;4(11):e0003888. doi: 10.1371/journal.pgph.0003888 (PMC11567621; doi:10.1371/journal.pgph.0003888)
Supplement: S2 Table — (DOCX) [file pgph.0003888.s002.docx]

**Occupational exposures in low- and middle-income countries: a scoping review**

Valentina Quintero Santofimio^1^, Andre FS Amaral^1,2^, Johanna Feary^1, 2^

^1^ National Heart and Lung Institute, Imperial College London, London, UK

^2^ NIHR Imperial Biomedical Research Centre, London, UK

**Corresponding author:** Valentina Quintero Santofimio, vq20@imperial.ac.uk

**Supplementary information**

**S2 Table.**  Search terms used within Web of Science and Medline (PubMed)

| **Subject heading** | **Search term** |
| --- | --- |
| Low middle income countries | Afghanistan OR afghanistan’s OR afghanistani OR afghani OR afghan OR afghans OR africa* OR albania OR albania’s OR albanian OR albanians OR algeria OR algeria’s OR algerian OR algerians OR angola OR angola’s OR angolan OR angolans OR antigua* OR barbuda* OR argentin* OR armenia OR armenia’S OR armenian OR armenians OR aruba* OR azerbaijan* OR bahrain* OR bangladesh* OR bengal* OR bangal* OR barbados* OR barbadian* OR bajan OR bajans OR belarus* OR belus* OR byelarus* OR byelus* OR belize* OR benin* OR dahomey OR bhutan* OR bolivia OR bolivia’s OR bolivian OR bolivians OR bosnia* OR herzegovin* OR botswan* OR batswan* OR bechuanaland* OR brazil OR brazil’s OR brazilian OR brazilians OR brasil OR brasil’s OR brasilian OR brasilians OR bulgaria* OR burkina* OR burkinese* OR upper-volta* OR burundi* OR urundi* OR cabo-verde* OR cape-verde* OR cambodia OR cambodia’s OR cambodian OR cambodians OR kampuchea* OR khmer* OR cameroon* OR cameroun* OR ubangi-shari* OR chad* OR chile OR chile’s OR chilean OR chileans OR china OR china’s OR chinese OR colombia OR colombia’s OR colombian OR colombians OR com ORo* OR com ORe* OR com ORian* OR mayotte* OR congo* OR zaire* OR costa-rica OR costa-rica’s OR costa-rican OR costa-ricans OR cote-d’ivoir* OR cote-d’ivoir* OR cote-divoir* OR cote-d-ivoir* OR iv ORy-coast* OR iv ORian* OR croatia* OR cuba OR cuba’s OR cuban OR cubans OR cyprus* OR cypriot OR cypriots OR czech* OR djibouti* OR french-somaliland* OR dominica* OR ecuad OR* OR egypt* OR united-arab-republic* OR el-salvad OR* OR guinea* OR equatoguinea* OR eritrea* OR estonia* OR eswatini* OR swaziland* OR swazi* OR swati* OR ethiopia* OR fiji* OR gabon* OR gambia* OR ((georgia* OR georgians) NOT (atlanta OR california OR fl orida)) OR ghana* OR gibraltar* OR greece* OR greek* OR grecian OR grecians OR grenada* OR grenadian* OR guam* OR guatemala* OR guyana* OR guiana* OR guyanese* OR haiti* OR hispaniola* OR hondura* OR hungary* OR india* OR indonesia* OR iran* OR iraq* OR isle-of-man* OR jamaica* OR j ORdan* OR kazakh* OR kenya* OR karabati* OR korea* OR kosovo* OR kosova* OR kyrgyz* OR kirgiz* OR kirghiz* OR laos OR lao OR laotian* OR latvia* OR lebanon* OR lebanese* OR lesotho* OR lesothan* OR lesothonian* OR basutoland* OR mosotho* OR basotho* OR liberia* OR libya* OR jamahiriya* OR lithuania* OR macedonia* OR madagasca* OR malagasy* OR malawi* OR nyasaland* OR malaysia* OR malay-federation OR malaya-federation OR malayan-federation OR maldives* OR maldivian* OR indian-ocean OR mali OR mali’s OR malian* OR malta OR malta’s OR maltese* OR micronesia* OR marshallese* OR kiribati* OR marshall-island* OR nauru OR nauran OR nauruans OR naurian’s OR mariana OR marianas OR palau OR paluan* OR tuvalu* OR mauritania* OR mauritan* OR mauritius* OR mexico* OR mexican* OR moldova* OR moldovia* OR mongol* OR montenegr* OR morocco* OR moroccan* OR ifni OR mozambique* OR mozambican* OR myanmar* OR burma* OR burmese OR namibia* OR nepal* OR new-caledonia* OR netherlands-antill* OR nicaragua* OR niger* OR oman OR oman’s OR omani OR omanis OR pakistan* OR palestin* OR gaza* OR west-bank* OR panama* OR paraguay* OR peru OR peru’s OR peruvian* OR philippine* OR philipine* OR phillipine* OR phillippine* OR filipino* OR filipina* OR poland* OR polish OR portugal* OR portuguese OR puerto-ric* OR romania* OR russia* OR ussr* OR soviet* OR rwanda* OR rwandese OR ruanda* OR ruandese OR navigator-island* OR pacific-island* OR polynesia* OR samoa OR samoan OR samoans OR sao-tome* OR santomean* OR saudi-arabia* OR saudi OR saudis OR senegal* OR serbia* OR seychell* OR sierra-leone* OR slovak* OR sloven* OR melanesia* OR solomon-island* OR norfolk-island* OR somali* OR sri-lanka* OR ceylon* OR saint-kitts OR st-kitts OR kittian* OR nevisian* OR saint-lucia* OR st-lucia* OR saint-vincent* OR st-vincent* OR vincentian* OR grenadine* OR sudan* OR surinam* OR syria* OR tajik* OR tadjik* OR tadzhik* OR tanzania* OR tanganyika* OR thai* OR timor-leste* OR east-timor OR tiese* OR togo OR togo’s OR togoles* OR tonga* OR trinidad* OR tobago* OR tunisia* OR turkiy* OR turkey* OR turk OR turks OR turkish OR turkmen* OR uganda* OR ukrain* OR uruguay* OR uzbek* OR vanuatu* OR new-hebrides* OR venezuela* OR vietnam* OR viet-nam* OR yemen* OR yugoslav* OR zambia* OR zimbabwe* OR rhodesia* OR arab-countr* OR arabic-countr* OR middle-east* OR global-south OR sahara* OR subsahara* OR magreb* OR maghrib* OR west-indies* OR caribbean* OR central-america OR central-america’s OR central-american OR central-americans OR latin-america OR latin-america’s OR latin-american OR latin-americans OR south-america OR south-america’s OR south american OR south-americans OR asia-central OR central-asia OR central-asia’s OR central-asian OR central-asians OR asia-northern OR north-asia OR north-asia’s OR north-asian OR north-asians OR northern-asia OR northern -asia’s OR northern -asian OR northern-asians OR asia-southeastern OR southeastern-asia OR southeastern-asia’s OR southeastern-asian OR southeastern-asians OR south-eastern-asia OR south-eastern-asia’s OR south-eastern-asian OR south-eastern-asians OR southeast-asia OR southeast-asia’s OR southeast-asian OR southeast-asians OR south-east-asia OR south-east asia’s OR south-east-asian OR south-east-asians OR asia-western OR west-asia OR west-asia’s OR west-asian OR west-asians OR western-asia OR western-asia’s OR western-asian OR western-asians OR europe-eastern OR east-europe OR east-europe’s OR east-european OR east-europeans OR eastern-europe OR eastern-europe’s OR eastern-european OR eastern-europeans OR developing-country OR developing-countries OR developing-nation OR developing-nations OR developing-population OR developing-populations OR developing-world OR less-developed-country OR less-developed-countries OR less-developed-nation OR less-developed-nations OR less-developed-world OR lesser-developed-country OR lesser-developed-countries OR lesser-developed-nation OR lesser-developed-nations OR lesser-developed-world OR under-developed-country OR under-developed-countries OR under-developed-nation OR under-developed-nations OR under-developed-world OR underdeveloped-country OR underdeveloped-countries OR underdeveloped-nation OR underdeveloped-nations OR underdeveloped-world OR middle-income-country OR middle-income-countries OR middle-income-nation OR middle-income-nations OR middle-income-population OR middle-income-populations OR low-income-country OR low-income-countries OR low-income-nation OR low-income-nations OR low-income-population OR low-income-populations OR lower-income-country OR lower-income-countries OR lower-income-nation OR lower-income-nations OR lower-income-population OR lower-income-populations OR underserved-country OR underserved-countries OR underserved-nation OR underserved-nations OR underserved-population OR underserved-populations OR under-served-population OR under-served-populations OR under-served-nation OR under-served-nations OR under-served-population OR under-served-populations OR deprived-country OR deprived-countries OR deprived-population OR deprived-populations OR high-burden-countr* OR high-burden-nation* OR countdown-countr* OR countdown-nation* OR poor-country OR poor-countries OR poor-nation OR poor-nations OR poor-population OR poor-populations OR poor-world OR poorer-country OR poorer-countries OR poorer-nation OR poorer-nations OR poorer-population OR poorer-populations OR poorer-world OR developing-economy OR developing-economies OR less-developed-economy OR less-developed-economies OR underdeveloped-economy OR underdeveloped-economies OR under-developed-economy OR under-developed-economies OR middle-income-economy OR middle-income-economies OR low-income-economy OR low-income-economies OR lower-income-economy OR lower-income-economies OR low-gdp OR low-gnp OR low-gross-domestic OR low-gross-national OR lower-gdp OR lower-gnp OR lower-gross-domestic OR lower-gross-national OR lmic OR lmics OR third-world OR lami-country OR lami-countries OR transitional-country OR transitional-countries OR emerging-economy OR emerging-economies OR emerging-nation OR emerging-nations) [tw] |
| **AND** | |
| Occupational setting | MeSH(Occupational exposures) |
| **AND** | |
| Quantitative occupational exposure measures | (Exposure measures OR quantitative measures OR exposure assessment OR levels of exposure OR exposure limits OR Threshold Limit Values. |
